# The Powerful Placebo Effect in Cough: Relevance to Treatment and Clinical Trials

**DOI:** 10.1007/s00408-019-00305-5

**Published:** 2019-12-13

**Authors:** Ron Eccles

**Affiliations:** Cardiff School of Biosciences, Cardiff University, Museum Avenue, Cardiff, CF10 3AX Wales UK

**Keywords:** Cough, Placebo effect, Voluntary control, Clinical trial, Taste, Excipients, Sweetness, Flavoring

## Abstract

Interest in the placebo effect of medicines has developed from the use of placebo treatments as controls in clinical trials into a whole new area of research around how placebos fit into a psychosocial model of therapeutics. The large placebo effect associated with cough medicines is both a problem and an opportunity for researchers: a problem for clinical trials on new actives as the active must beat the large placebo effect, and an opportunity for harnessing the placebo effect to produce effective cough medicines without any pharmacologically active ingredient. This review discusses the mechanisms associated with the placebo effect of cough medicines and distinguishes between a ‘perceived placebo effect’ and a true ‘placebo effect’. The efficacy of sweeteners in cough syrups is discussed as well as viscosity, mucoadhesion, and flavoring. The complexity of modern cough medicines is demonstrated by an example of a medicine which contains one active ingredient, and eighteen excipients which provide a complex and intense sensory experience to enhance the placebo effect and complement the pharmacological activity of the medicine.

## Introduction

Cough is one of the most common and distressing of symptoms, yet at present, there is little to offer patients to relieve cough apart from cough syrups which are effective because of a powerful placebo effect [1, 2]. Codeine was once deemed a powerful antitussive for all types of cough because of its presumed depressant effect on cough control at the level of the brainstem but doubts have been raised on its efficacy and it is no longer considered a ‘gold standard’ antitussive [3]. Much progress is being made on the peripheral mechanism of cough at the level of sensory nerves in the airway but clinical trials on some of the best candidates for a new cough medicine are proving difficult because of side effects [4]. The placebo effect in cough therapy is both a gift and a problem to those working on treatments for cough. It is a gift because cough is so susceptible to a placebo effect with one review reporting that up to 85% of the efficacy of cough medicines is due to a placebo effect [5]. It is also a problem to researchers as with such a large placebo effect it is difficult to demonstrate that any new cough medicine is superior to placebo treatment in cough clinical trials. The magnitude of the placebo effect in the treatment of cough with inhaled steroids has recently been highlighted and the authors conclude that clinicians should be cautious about attributing the response of patients on inhaled steroids to anything more than a placebo effect [6]. This review will discuss the significance of the placebo effect in the development of new cough treatments and its importance in cough clinical trials.

## What is a Placebo and a Placebo Effect?

The term ‘placebo’ originates as a Latin term and means ‘I shall please’ [7]. Before the introduction of codeine as a therapy for cough in the nineteenth century [8], all cough medicines could be considered as placebo treatments that were meant to please patients even though physicians and patients may have believed that they had active properties above that of a placebo treatment. An early edition of the Merck’s manual [9] lists 61 treatments for cough, including carbolic acid, alcohol, cannabis, indica, creosote, morphine, potassium bromide, sandalwood oil, and zinc sulfate, and describes cod-liver oil as the most useful of all remedies in cough. All of these cough medicines are likely to have had some beneficial placebo effect and reduced the severity of cough, and that is why they were regularly prescribed by physicians and regularly pleased patients. When patients are given any cough medicine, they usually feel better and the severity of their cough is reduced but it is difficult to separate out any pharmacological effect of the medicine from any placebo effect unless the treatment is compared with a placebo treatment in a double-blind randomized clinical trial.

### Perceived and True Placebo Effects

The efficacy of any cough medicine depends on two factors: firstly the efficacy of the pharmacologically active medicine such as codeine or dextromethorphan and secondly the placebo effect of the treatment. When discussing the placebo effect, it is important to separate the ‘perceived placebo effect’ from the ‘true placebo effect’. This distinction was first put forward by Ernst in 1995 [10] and was later extended to explain the placebo effect of cough medicines [5, 11].

**Fig. 1:**
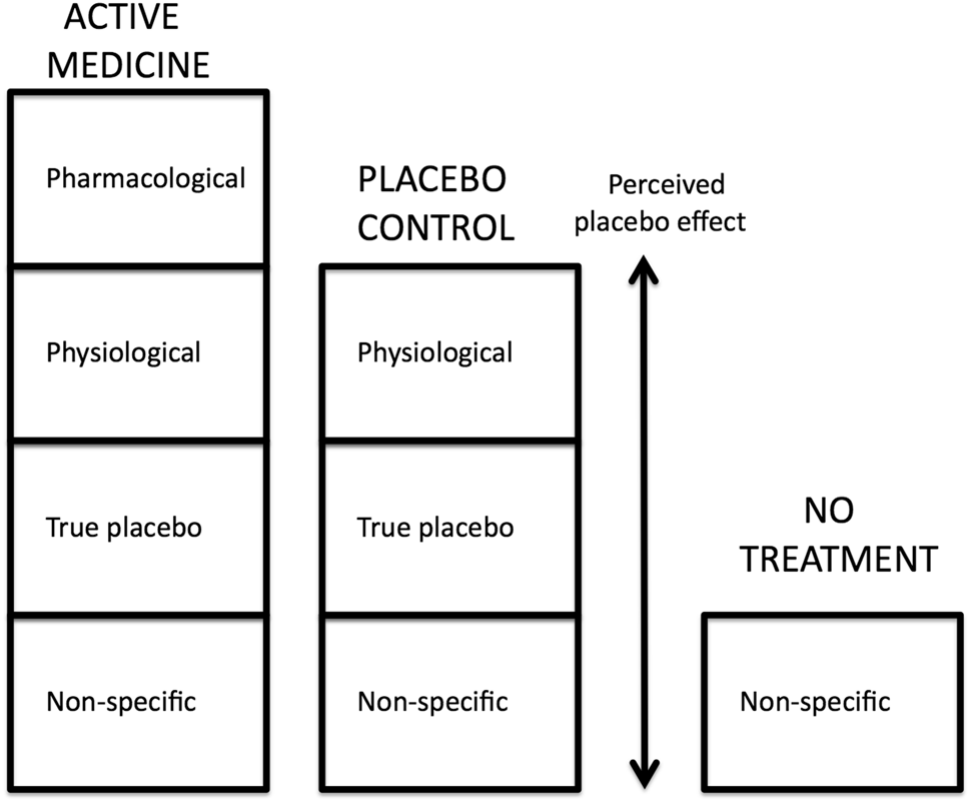
Efficacy of any cough syrup medicine in a clinical trial consists of four components. Pharmacological related to the efficacy of the active ingredient. Physiological related to the demulcent effect. True placebo related to the sensory impact of the medicine and belief of the participant abut efficacy. Non-specific related to natural recovery. The total efficacy of the placebo control treatment is the PERCEIVED PLACEBO EFFECT and it includes the TRUE PLACEBO EFFECT and the non-specific effect of natural recovery over the time period of the trial

The perceived placebo effect is the total response to placebo treatment seen in a clinical trial, and the perceived placebo effect is made up of three components as illustrated in Fig. 1. The ‘physiological effect’ of a cough treatment is the effect due to the taste of the medicine and is related to stimulation of salivation and mucus secretions that soothe and lubricate the airway, and may also be related to a specific effect of sweet taste as discussed below. The physiological effect of a cough medicine is utilized in cough syrups as a major component of their efficacy, and this physiological effect is not present or minimal in cough medicines formulated as a tablet or capsule. The ‘non-specific’ components of any treatment are related to factors such as natural recovery of the patient, and regression to a mean value of cough severity, as patients are generally recruited into a clinical trial with severe symptoms and these measures can only regress towards a mean value. The true placebo effect can be considered as the psychological effects of the treatment that lead to a reduction in cough severity. The true placebo effect was first of interest in clinical trials but interest in this phenomenon has evolved into a psychosocial model which incorporates a general interaction of the patient to their environment and how they respond to psychological mechanisms such as conditioning, expectation, reward, and anxiety reduction, and how these can be modulated by desire, motivation, and memory [12].

**Fig. 2:**
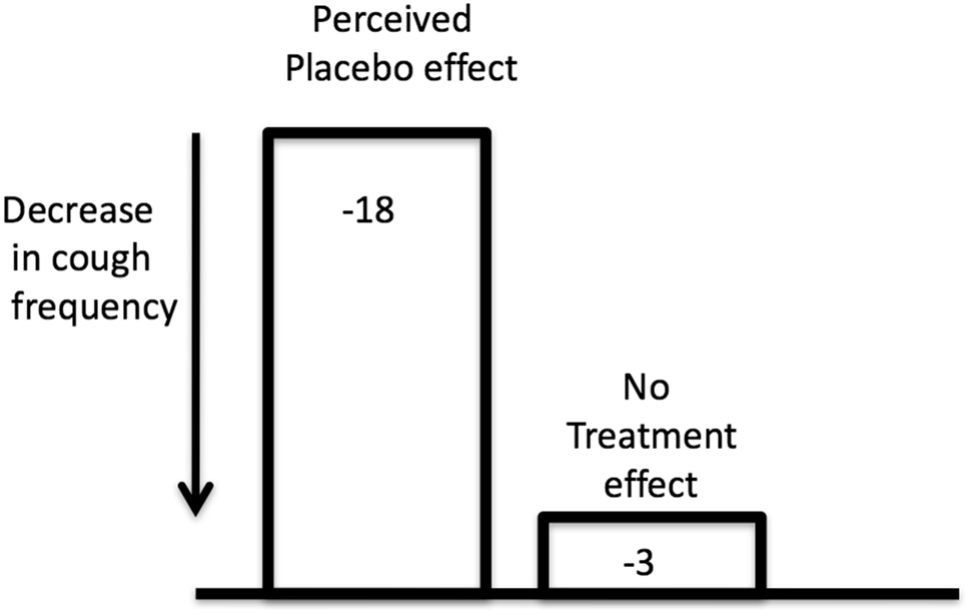
Change in median cough frequency over 15 min period after treatment with a placebo medicine (vitamin E capsule, *n* = 27) or no treatment (*n* = 27). Both groups took 50 ml of water. The small reduction in cough frequency seen in the no treatment group may be related to a demulcent effect of water. The magnitude of the true placebo effect can be estimated by subtracting the no treatment effect from the perceived placebo effect

As can be seen in Fig. 1, the component of the true placebo response is part of the perceived placebo response that is measured in clinical trials. In order to determine the magnitude of the true placebo response it is necessary to conduct a clinical trial with a ‘no treatment’ group and subtract the response of the no treatment group from the response of the perceived placebo group. The physiological response, which is also contained in the perceived placebo response, can be minimized by using a tablet formulation as the treatment rather than a syrup. Clinical trials rarely contain a no treatment group and only one study on acute cough associated with common cold has been performed with this design [13]. In this study the placebo treatment consisted of a capsule containing vitamin E, which over the short duration of the measurement period for cough (15 min) would not have been absorbed to have any effect on cough. The effect of placebo treatment on cough frequency is illustrated in Fig. 2 which shows a marked reduction in cough frequency compared to no treatment. The small reduction in cough frequency associated with no treatment could be due to a demulcent effect as both groups were given 50 ml of water to drink even though the no treatment group did not need this water to swallow any capsule. The difference between the placebo treatment group and the no treatment group in this study gives us a measure of the true placebo effect and it indicates and demonstrates its significance in cough therapy.

## What Makes a Good Placebo Cough Medicine?

If placebo treatments are effective at treating cough why not enhance the placebo effect and use a placebo treatment as a commercial cough medicine? Issues around this approach are firstly those concerning the patient’s perception of the medicine and secondly the regulatory environment and claims that can be made for the medicine. Patients will not purchase or believe in a medicine that they know has no active component or which is referred to as a placebo, and regulatory authorities will not allow claims for cough medicines composed only of flavoring and excipients. However, with publicity around clinical trials testing honey as a cough medicine [14–16] and negative reports about conventional antitussives such as dextromethorphan [17, 18], the public has become more convinced about the efficacy of simple linctus medicines containing honey and glycerol and these simple linctuses are now marketed with honey, glycerol, and sugar declared as active ingredient [19]. The great advantage of this type of medicine is that they are supported by pediatricians as safe for children and they can be dosed frequently without any major risk of side effects. In developing a powerful placebo cough medicine the following components of the medicine need to be considered.

### Taste

Look in any pharmacy for a cough medicine and you will see that the great majority of those on sale are formulated as sweet viscous syrups. Viscous syrups formulated as a cough medicine are known as a linctus. This formulation of a cough medicine has evolved over thousands of years and is probably related to the properties of the first cough medicine, natural honey. Natural honey has been used as a cough medicine for thousands of years and it is still popular as a cough treatment today [20]. Natural honey varies widely in its composition, color, and taste and it contains about 200 substances, including amino acids, vitamins, minerals, and enzymes, but it primarily contains sugar and water [20]. Sugars such as glucose, fructose, maltose, sucrose account for 95–99% of honey dry matter [20]. Honey was the first source of a sweetener for cough medicines but from the fifteenth century onwards sugar cane and sugar beet started to provide a much cheaper and more readily available source of sugar for cough medicines especially as inverted sugar syrup, which is often referred to as artificial honey, because of its sweetness and viscosity [21]. Natural honey has been shown to be just as effective as conventional pharmacological treatments [14, 22, 23], but it is likely to be the sweet taste of honey that is the major factor in its efficacy rather than any specific pharmacological property of the honey. A recent study comparing buckwheat honey with a matched Golden Syrup sweet placebo on treatment of nocturnal cough in children has reported that honey is superior to matched sweet placebo [16]. However, the blinding of the study was not confirmed with any patient questionnaire or other means, and therefore the results could still be explained as comparing one sweet placebo against another sweet placebo, rather than honey having any specific effect on cough.

**Fig. 3:**
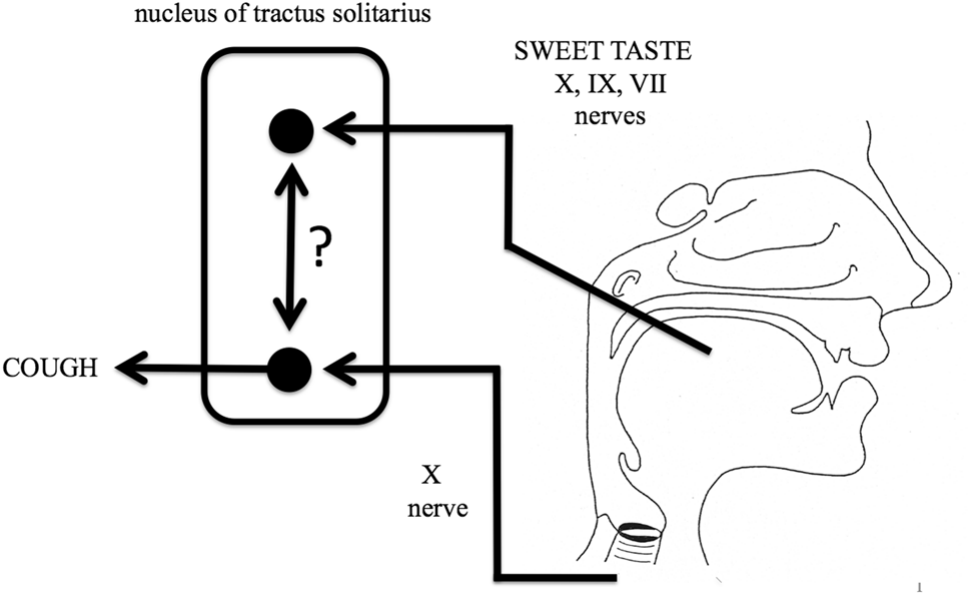
Gustatory effects on cough. Gustation is mediated by branches of the facial (VII), glossopharyngeal (IX), and vagus (X) cranial nerves that supply the taste buds of the tongue, these gustatory nerves relay in the nucleus tractus solitarius (NTS) in the brainstem. The NTS also serves as a relay for the vagal nerve fibers (X) that mediate the cough reflex. It is proposed that there may be some interaction (?) between these gustatory and cough pathways in the NTS that influences cough, perhaps by the mediation of endogenous opioids

Sweet cough syrups have been proposed to act as antitussives by sweet taste modulating the activity the nucleus tractus solitarius at the level of the brainstem as illustrated in Fig. 3 [2]. This hypothesis was tested using capsaicin induced cough in healthy subjects as a cough model and sweet but not bitter taste was shown to increase cough reflex thresholds [24, 25]. Sweet taste may therefore have a specific antitussive activity rather than just be a pleasing taste for the patient. This may also explain the traditional use of liquorice in the treatment of cough as the active constituent of liquorice, glycyrrhizin, is 20–50 times the sweetness of glucose weight for weight [26].

A sweet taste may be only one way of enhancing the placebo effect of a cough medicine as the placebo effect may also be related to the organoleptic sensory impact of the medicine, and for some patients, an unpleasant taste of a medicine indicates a powerful medicine [27]. Capsicum flavor provides a powerful taste to some cough medicines and may also act as a gustatory stimulus to increase mucus secretions in the airway [28, 29].

### Cooling, Warming, and Tingling Effects

Cooling and warming agents are often added to cough medicines to increase the sensory impact of the medicine and perhaps increase its placebo effect. Capsicum is a flavoring agent that is an extract of peppers and contains capsaicin which interacts with oral sensory trigeminal nerve endings to activate TRPV1 receptors to give a sensation of burning irritation and warmth [30]. Capsicum flavor gives a powerful hot taste to lozenges such as ‘Fisherman’s Friend’ which have been marketed for over one hundred years to treat respiratory conditions such as cough [31, 32]. Menthol is included in many cough syrups and lozenges to give a cool sensation and is referred to as either a flavoring agent or an active ingredient. Menthol acts on the TRPM8 receptor on sensory nerves in the oral cavity and airway to give a cool sensation [33–35]. Apart from providing a sensory impact to a cough medicine menthol may also have some specific antitussive properties [24, 36–38] and menthol is recognized as an antitussive by regulatory authorities such as the US Food and Drug Administration.

Capsicum and menthol have a long history as warming and cooling agents but they are now often replaced in cough medicines by new flavoring excipients such as cooling flavor 539692T and hot mix flavor 538842T which are dissolved in propylene glycol and manufactured and marketed by specialized flavoring companies.

The placebo effect is influenced by the sensory impact of the cough medicine and this sensory impact can be enhanced by excipients that give a tingling sensation. Tingling flavor 538723T is added to some cough medicines to provide this tingling sensation.

### Viscosity

As mentioned above, most cough medicines are formulated as viscous syrups. The viscosity of the medicine may have a sensory impact that enhances a placebo effect in two ways. Firstly, a viscous thick syrup may be perceived by the patient as a more powerful medicine than a watery medicine. Secondly, a viscous medicine will tend to stick to the oral mucosa and esophagus and provide a more prolonged sapid stimulus than a watery medicine. Viscous syrups such as honey provide a more prolonged sweet taste than sugar water as they tend to stick to the oral mucosa and teeth. High concentrations of glucose and other sugars as well as inverted sugar will increase the viscosity of a cough medicine. Glycerol is a common component in cough medicines and it increases both the viscosity and the sweetness of the medicine [39]. The viscosity of cough medicines may be increased by addition of excipients such as carboxymethylcellulose sodium and carbomer, which can also act as coating agents to stick to the oral mucosa and prolong the sensory impact of flavors in the medicine [40].

### Color

The placebo effect of medicines such as tablets is influenced by the color of the tablet with red yellow and orange associated with a stimulant effect and blue and green associated with a tranquilising effect [41, 42]. Cough medicines are often colored and the most common colors are brown and red. The brown color often relating to the presence of a caramel flavor and the red color related to the addition of Ponceau 4R (E124). The brown and red colors may impart a sense of strength to the placebo effect although no published research has been found on the effect of color on the placebo effect of cough medicines.

### Odor

No information has been found in the literature on the use of odors to enhance or trigger placebo effects in cough medicines, but since odors have a great capacity to evoke memory and to influence behavior and emotion [43], it is likely that the odor of a cough medicine will influence the placebo effect of the medicine. Odor-evoked memories are especially visceral because of the neuroanatomy of olfaction and they evoke more emotional and evocative recollections than memories triggered by any other sensation [43]. Odors may be an under utilized tool in enhancing the placebo effect of cough medicines. Most cough medicines have a medicinal odor or a menthol odor and theses odors may trigger memory and influence the placebo effect. Large placebo effects have been reported for odors in aromatherapy, and a coffee-like scent which contains no caffeine can elicit a placebo effect and increase arousal [44, 45]

### Belief

A conscious belief in the efficacy of a cough medicine is probably the most important factor in determining the magnitude of the true placebo effect. The factors listed above—taste, cooling, warming, tingling, viscosity, and color—all contribute to the sensory impact of the medicine and influence the perceived placebo effect, but if the patient does not believe in the efficacy of the medicine the sensory impact will have little if any influence on the true placebo effect. The sensory impact of the treatment can still provide a demulcent and soothing effect through stimulation of salivation and mucus production but unless the patient believes in the efficacy of the medicine it will not have a true placebo effect.

The role of belief in the placebo effect has been discussed in detail by Evans in the book “Placebo. The Belief effect” [46], and a full discussion on the influence of belief on the placebo effect is beyond the remit of this review which is focussed on the role of belief in the efficacy of a cough medicine.

**Fig. 4:**
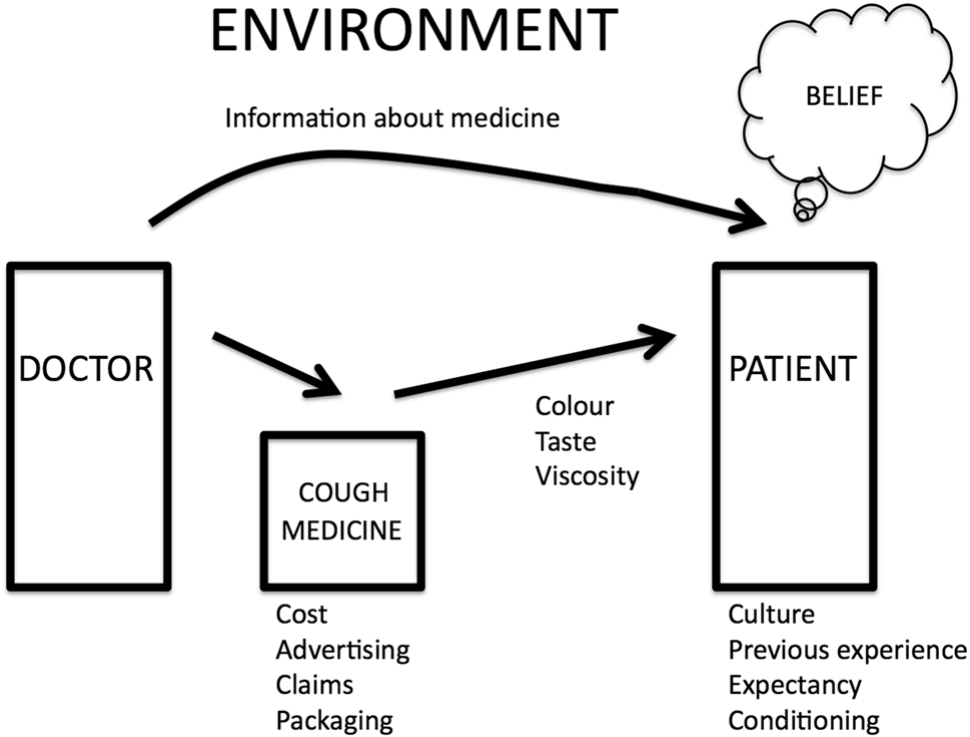
Factors influencing patient belief about the efficacy of a cough medicine, in this example illustrating doctor–patient interaction

Belief in the efficacy of a cough medicine is influenced by many different factors which have been described as the “psychosocial context around the therapy” [12]. Some of these factors are illustrated in Fig. 4. Belief about any cough therapy will first depend on the context of the therapy: is the patient consulting a healthcare practitioner such as a doctor, nurse or pharmacist in order to receive therapy or are they purchasing an over-the-counter (OTC) medicine from a supermarket or pharmacy outlet on their own initiative to obtain a cough therapy? In the first instance, the doctor–patient relationship and belief in the expertise of the practitioner will influence belief in the prescribed therapy, and in the second instance, purchase of an OTC cough medicine will be influenced by factors such as advertising claims, packaging, cost, previous experience, personal recommendations. The environment in which the therapy is prescribed or purchased will also influence belief as a prescribed therapy delivered in a famous research hospital by an eminent physician at great expense may influence belief about the efficacy of the therapy more than the belief associated with picking up a cheap OTC cough medicine from a supermarket. The placebo and the doctor–patient relationship has been discussed in detail by Benedetti [47] in terms of a new physiology which explores the relationship between belief and neurobiological phenomena.

## Does the Placebo Effect Confound Cough Clinical Trials?

The placebo effect may confound clinical trials on cough medicines because the placebo effect is of such a great magnitude that it leaves little room for any pharmacologically active substance to influence cough. This understanding has come from a review on acute cough which quantified the placebo effect as contributing up to 85% of the efficacy of any treatment, with the pharmacologically active ingredient only contributing 15% of the benefit of the cough medicine [5]. However, it is important to take into consideration two factors when considering the large magnitude of the placebo effect described in the review. Firstly, all the studies used in the review to calculate the placebo effect were conducted on acute cough, and spontaneous recovery may make a large contribution to the perceived placebo effect in these studies. Secondly, the studies described in the review were mainly conducted using dextromethorphan and codeine as actives, and the efficacy of both of these antitussives on acute cough has been challenged [3, 17, 48]. The large perceived placebo effect reported by the review may be due to spontaneous recovery of subjects and the weak pharmacological efficacy of the actives. It is also important to consider that much more information is needed about the magnitude of the placebo effect in clinical trials, as in a meta-analysis on the placebo effect in clinical trials on analgesics it has been shown that there is a great variability in the magnitude of the placebo effect [49, 50]. With only one review article Eccles [5] assessing the magnitude of the perceived placebo effect in cough clinical trials, involving only eight clinical trials on cough, it is not possible to properly estimate the magnitude of any true or perceived placebo effect in a cough clinical trial. It is not possible to determine if cough clinical trials are confounded by a large placebo effect and at present the main issue appears to be a lack of efficacy of the active compounds tested as antitussives.

### Voluntary Control of Cough

Cough is an unusual symptom in that it is under voluntary control [51]. Healthy subjects can cough and their cough is indistinguishable from a cough associated with an upper respiratory tract infection. Similarly cough associated with upper respiratory tract infection can be voluntarily suppressed [52]. This voluntary control of cough can confound clinical trials that use any sort of cough count as an endpoint, as if subjects believe they are receiving an active cough medicine then they may be able to alter their cough response in accord with their beliefs and cough less. One could argue that any voluntary control of cough is not a placebo effect as the control of cough can be achieved without any placebo treatment but ‘belief’ is a major factor in determining the magnitude of any true placebo effect and it is difficult to separate between a conscious voluntary control of cough and the unconscious response to a placebo treatment. In the past, clinical trials on cough medicines have focussed on cough counts and the severity of cough as perceived by the patient but more recently there has been interest in ‘the urge-to-cough’ which is dependent on a sensation of airway irritation and which precedes any cough [53]. The urge-to-cough has been shown to be influenced by placebo treatment, indicating that whatever measure of cough is used in clinical trials it is still subject to a placebo effect [54].

### Kinetics and Dynamics of Placebo Effect

A pharmacologically active cough medicine such as dextromethorphan will have pharmacokinetic and pharmacodynamic properties, and studies on its efficacy as a cough medicine will take these properties into consideration when deciding on which time points after dosing to measure its antitussive activity. The physiological effects of a placebo treatment such as a sweet cough syrup will also exhibit similar properties to a pharmacologically active medicine with characteristics such as time and intensity of peak response and rate of decline of response, which can generate pharmacokinetic parameters. There is little if any literature on the kinetics and dynamics of the placebo effect, although this topic was raised as an important aspect in clinical trials by Weiner and Weiner [42]. The true placebo effect, which is initiated by belief in the efficacy of the medicine, can also be considered to have pharmacokinetics even though there is nothing to be absorbed, distributed, metabolized or excreted, as the effects in the brain are eventually related to release and actions of neurotransmitters which do have pharmacokinetic properties [42]. At present the lack of knowledge about the pharmacokinetic and pharmacodynamics of the placebo effect is a possible confounding factor in the design of clinical trials on cough medicines.

### What Information Should be Given to the Patient About Placebo Treatment?

The patient information leaflet (PIL) is an important component of the informed consent process of admitting patients into a clinical trial as the patient must be given all the relevant information about the test therapy in order to make an informed opinion as to whether or not to participate in the clinical trial. The PIL usually describes possible benefits and adverse effects and risks associated with the active cough therapy but information about the placebo treatment is usually brief, incomplete and sometimes inaccurate [55]. The words used to describe the placebo treatment are often a description of a “dummy” pill or “inactive control medicine” that looks and tastes like the “real” medicine. No studies have been found in the literature on the information given in PIL in cough clinical trials. A study on the PIL information about placebos in clinical trials on acupuncture reports that how participants are told about placebos may influence the blinding of the study and the study outcome by influencing patient expectation [56].

Studies on the true placebo effect of cough therapy are often confounded by ethical considerations to tell the truth about the treatments and the purpose of the study. In a study to determine the magnitude of the true placebo effect of cough therapy in a clinical trial, vitamin E was used as a placebo therapy but the term “placebo” was not used in the PIL, which stated that the “The study is designed to investigate the effect of vitamin E on cough associated with the common cold” [13]. The effects of vitamin E treatment (placebo) on cough were compared to a “no treatment” group in order to determine the true placebo effect of therapy [13]. The study provided new information about the magnitude of the true placebo effect of cough therapy, but it can be criticized as using deception in the PIL, as the true aim of the study was not given in the PIL. Deception in the research on the placebo effect has been discussed in detail in a review article [57] and the authors discuss the use of “authorised deception” in placebo research where the participants are informed in the PIL that some form of deception may be used in the research [57].

Since placebos do have beneficial effects, a case has been made that much more information should be provided in the PIL about the positive benefits of placebo therapy and the placebo effect, and that lack of knowledge about the placebo effect in the PIL breaches the ethical obligations of the researchers [58]. A case is made that there is substantial knowledge about the effects of placebo treatment on the brain and especially on the analgesic effects of placebos but that this knowledge is not reflected in the informed consent procedures [58]. Informing clinical trial participants about the health benefits of placebos may be good clinical practice as regards a more accurate and comprehensive PIL, but it may influence participant expectations about the therapies used in the trial and bias the outcome of the trial. Exactly what information should be given about placebo treatments in PIL remains a controversial area for clinical researchers.

## How Can the Placebo Effect be Utilized in the Development of New Cough Medicines?

Cough medicines have evolved over centuries to maximize the placebo effect by being formulated as sweet viscous syrups and this evolution is still in progress today as pharmaceutical companies utilize new flavors and sensations to increase the impact of their OTC cough medicines. There is an ongoing research effort to develop new antitussive medicines and most of these involve clinical trials on actives in tablet form [4, 59]. However, if these new active agents are eventually used commercially for the treatment of cough, the efficacy of the cough medicine would be greatly enhanced by formulating the medicine as a sapid sweet syrup in order to utilize a large placebo effect of treatment in addition to any pharmacological effect of the medicine.

**Fig. 5:**
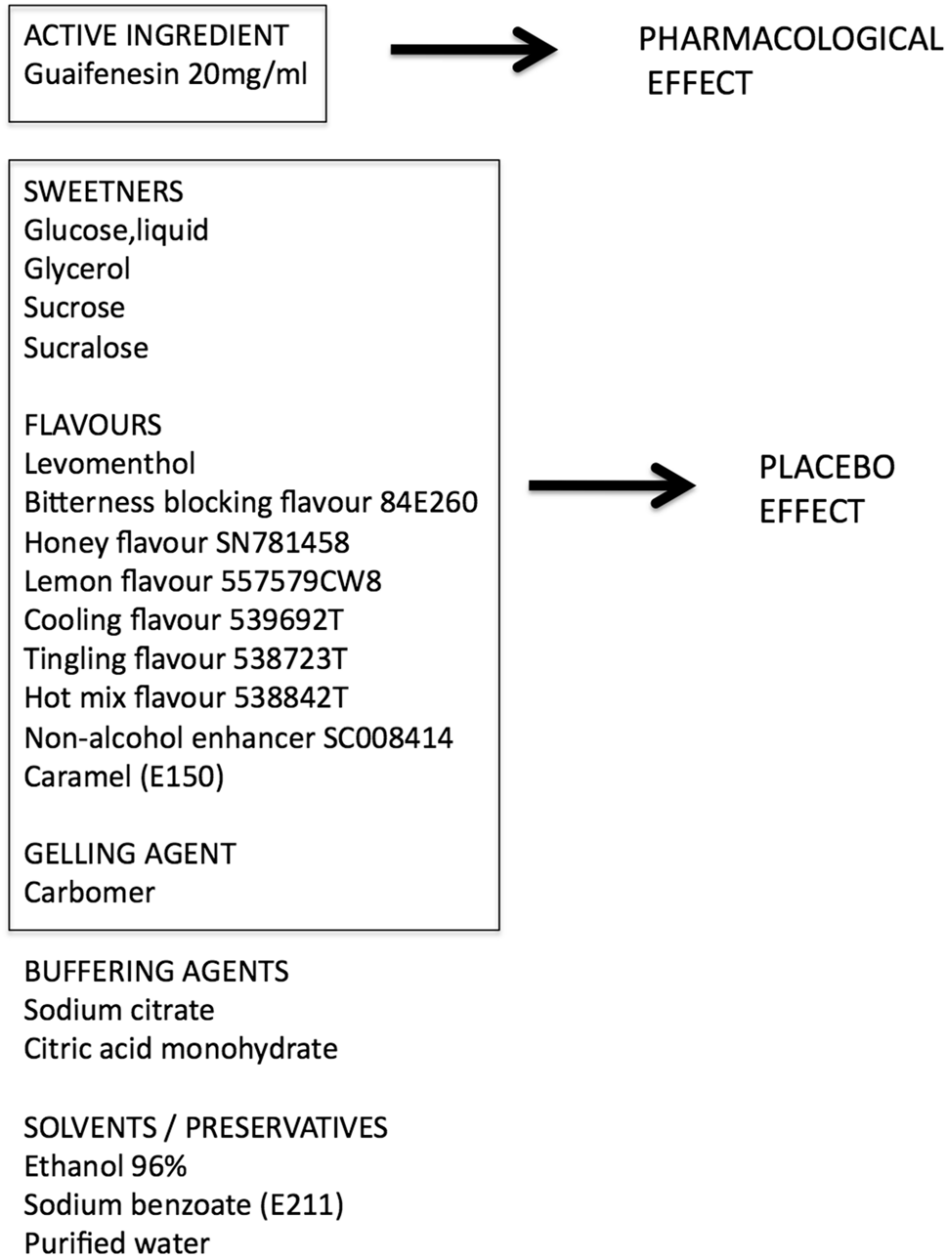
List of ingredients of modern over-the-counter cough medicine (Benylin Mucus Cough Max Honey & Lemon Flavor 100 mg/5 ml Syrup) as detailed in summary of product characteristics (SmPC) of medicine

The formulations of OTC cough medicines have evolved to provide powerful placebo effects by utilizing the effects of excipients and flavors. The composition of a current OTC cough medicine [60] is illustrated in Fig. 5, and it demonstrates that the ingredients provide a complex mix of sweeteners and flavors that enhance the placebo effect of the medicine. In this medicine the sweetness is provided by not one but four ingredients (glucose, glycerol, sucrose, and sucralose). The sugar content (glucose and sucrose) is 898 mg/ml which translates into a dose of 36 g of sugar a day, and the sweetness is further enhanced with the inclusion of glycerol and sucralose (sucralose has a sweetening power of 300–1000 times that of sucrose [40]. The viscosity of the syrup is increased by the presence of glycerol and carbomer, and the carbomer also provides some mucoadhesive properties to the syrup to bind it to the oral mucosa. The nine flavors provide an intense and complex sensory impact with honey, lemon, and caramel flavors and cooling and warming sensations. This example of an OTC cough medicine demonstrates how far these medicines have evolved to enhance the placebo effect and complement any pharmacological effect of the active ingredient for the benefit of the patient.

## Conclusion

The first cough medicines such as honey had only a placebo effect but they were effective medicines that pleased patients. In order to determine the pharmacological activity of medicines, the double-blind placebo controlled clinical trial method was developed and many cough medicines were shown to have no more efficacy than a placebo treatment. The perceived placebo effect in cough studies has been shown to consist of three components: a physiological effect related to the taste of the medicine, a non-specific effect related to natural recovery, and a true placebo effect related to belief in the efficacy of the medicine. The magnitude of the perceived placebo response has been shown to be up to 85% in some cough clinical trials, and this may confound the conduct of cough clinical studies but it is an advantage in developing OTC cough medicines that can utilize new flavors and sensations to enhance the placebo effect.
